# Stress overload, influencing factors, and psychological experiences of nurse managers during early stages of the COVID-19 pandemic: a sequential explanatory mixed method study

**DOI:** 10.3389/fpsyg.2023.1187433

**Published:** 2023-06-30

**Authors:** Yundan Jin, Feifei Cui, Rongting Wang, Shuainan Chen, Lina Hu, Meiqi Yao, Haiying Wu

**Affiliations:** ^1^Department of Nursing, The Affiliated Dongyang Hospital, Wenzhou Medical University, Dongyang, China; ^2^School of Health Management, Jilin Medical University, Jilin, China; ^3^Department of Nursing, School of Medicine, The Second Affiliated Hospital, Zhejiang University, Hangzhou, China

**Keywords:** COVID-19, nurse managers, stress overload, influencing factors, work family support, psychological experiences

## Abstract

**Background:**

Healthcare systems had an exceptionally difficult time during the early COVID-19 pandemic. Nurse managers in particular made enormous contributions to ensuring the safety of patients and front-line nurses while being under excessive psychological stress. However, little is known about their experiences during this time.

**Objective:**

The aim of this study was thus to assess the level of stress overload and psychological feelings of nurse managers during the early COVID-19 pandemic.

**Methods:**

A mixed methods sequential explanatory design study with non-random convenience sampling was performed, following the STROBE and COREQ checklists. The study was conducted at the Affiliated Dongyang Hospital, Wenzhou Medical University, with data collected from six provinces in southern China (Zhejiang, Hubei, Shanghai, Jiangsu, Hunan and Jiangxi) during March 2020 and June 2020. A total of 966 nurse managers completed the Stress Overload Scale and Work-Family Support Scale. In addition, a nested sample of nurse managers participated in semi-structured face-to-face interviews. The data were then analyzed using qualitative content analysis, Pearson correlation, and multiple linear regression.

**Results:**

The quantitative results showed that nurse managers experienced a moderate level of stress load. There was a significant negative correlation between work-family support and stress load (*r* = −0.551, *p* < 0.01). Concerns about protecting front-line nurses and work-family support were the main factors affecting the stress load, which accounted for 34.0% of the total variation. Qualitative analysis identified four main thematic analyses that explained stress load: (1) great responsibility and great stress, (2) unprecedented stress-induced stress response, (3) invisible stress: the unknown was even more frightening, and (4) stress relief from love and support. Taken together these findings indicate that concern about protecting front-line nurses and negative work-family support of nurse managers were the main factors causing stress overload.

**Conclusion:**

Implementing measures focused on individual psychological adjustment combined with community and family support and belongingness is one potential strategy to reduce psychological stress among nurse managers.

## Introduction

The rapidly spreading and life-threatening nature of the COVID-19 pandemic placed unprecedented pressure on patients, healthcare workers, and society (Liu X. et al., 2020; Jackson and Nowell, 2021). Prior studies have highlighted the unique contributions of clinical nurse managers during the early stages of the pandemic on three groups: patients, organizations, and nurses (Mamais et al., 2022). Nurse managers perform nurse management functions, and their planning, organization, leadership, and control abilities play a vital role in efficient use of human, material, and financial resources. During the COVID-19 pandemic, clinical nurses, as the main front-line workers, were under enormous stress: not only did they face infection risk and excessive workload, they also needed to continue to fight in the absence of human resources, equipment, and clear guidance (Liu Q. et al., 2020). As a result, front-line nurses are reported to have faced many health-related adverse outcomes, such as anxiety, depression, insomnia, fear, and poor mental resilience (Han et al., 2020; Kackin et al., 2021; White, 2021). However, the roles and responsibilities of nurse managers differ from those of clinical nurses in that they have a higher level of leadership and coordination of activities (Jackson and Nowell, 2021). Under pandemic-related challenges, nurse managers had additional responsibilities for resource management, personnel, and equipment deployment and were prone to huge stress overloads that induce psychological stress responses. Therefore, it can be speculated that nurse managers experienced similar reactions as front-line nurses but with more severe experiences of stress and discomfort.

Strong psychological stress resilience and support from work and family are extremely important for clinical nurse managers. However, it is not known whether supports that benefitted clinical nurses during COVID-19 were equally useful for nurse managers. There is a dearth of mixed studies on stress overload, psychological experience, and influencing factors among nurse managers. Thus, this study aimed to fill this research gap to provide a reference for the optimization of clinical intervention decision-making and program adjustment.

## Background

According to Stress Theory, individuals can experience emotional, physiological, and behavioral responses when facing various stress and challenges. These responses are considered to be a stress reaction, resulting from a demand on an individual’s resources that exceeds their coping abilities. The theory emphasizes the interplay between an individual’s behavior, personal characteristics, and environment, providing an explanation for why some people may be more susceptible to stress than others in the same situation (Lazarus and Folkman, 1984). Psychological theories of stress emphasize perception over physiology. Pathogenic forms of stress arise from specific situations in which demands outweigh resources; they are seen as challenging but not destructive if the demands are assessed as being within a person’s capabilities, and only threatening when the demands exceed coping resources, leading to physical and mental dysfunction (Amirkhan, 2012). The term “stress overload” is used to describe a persistent state of being overwhelmed by demands that increases susceptibility to disease (Lunney, 2006). The period from March to September 2020 was a time of a surge in infections with novel coronavirus (COVID-19), which was uneven and affected certain populations more severely. Media reports had provided explanations for the vulnerability and susceptibility of specific populations, but psychological risk factors have been largely ignored (Amirkhan, 2021). Multiple medical and psychological theories point to a psychosocial factor-stress-as being equally important to the etiology of disease (Lunney, 2006).

Negative emotions, anxiety, depression, and stress associated with caring for COVID-19 patients were prevalent among front-line staff in the early stages of the outbreak, according to quantitative and/or qualitative studies (Han et al., 2020; Moghaddam-Tabrizi and Sodeify, 2021; White, 2021; Heydarikhayat et al., 2022). Recent studies mainly focus on the factors influencing the stress load of clinical front-line nurses (D'Emeh et al., 2021). Sharifi et al. (2022) found that most nurses providing care for COVID-19 patients experienced a severe stress response, with prevalence rate of moderate to severe depression, anxiety, and stress of 43.7, 73, and 24%, respectively. Nurse managers may have experienced these feelings as well. Sun et al. (2020) found that the negative emotions such as fatigue, discomfort, and helplessness in the early stage of care for patients with COVID-19 were related to the high intensity of work, fear, and anxiety, as well as the care for patients and their families. Mo et al. (2020) found that nurses working in Wuhan, China, showed a high stress load, especially nurses who were single parents and/or had extended working hours.

During the pandemic in Singapore, nurse managers had the additional task of caring for employees and their psycho-emotional well-being, besides routine management (White, 2021). A study investigating the role of nurse managers during the pandemic found that workplace conditions, such as organizational support, organizational preparedness, workplace safety, and access to supplies and resources, were associated with higher scores related to adverse mental health outcomes (White, 2021; Al Sabei et al., 2022; Kangarlou et al., 2022). Stress Theory suggests that family and social support also plays a critical role in alleviating the negative impact of stress and helps individuals better cope and adapt to adversity (Lazarus and Folkman, 1984). Strengthening support among nurses could reduce the impact of work-related stress on health. In terms of more tangible resources, some people were blessed with material comfort or social support, while others were ill-equipped to cope with the economic and emotional impact of the pandemic. However, people who were disadvantaged in both areas were most likely to experience stress overload, facing greater demands from the pandemic with fewer resources (Amirkhan, 2021). Yu et al. (2020) found that stress from work–family conflict was positively correlated with nurse managers’ overall well-being. Work–family conflict is recognized as a major source of stress and associated with psychological stress and job dissatisfaction in certain populations, especially during the pandemic. Nurses managers were more involved in nursing management and quality control than general nurses and experienced more serious work–family conflict in their work-family roles than general nursing staff. Nurse mangers are reported to experience significantly higher rates of work–family conflict (Yu et al., 2020). Li and Wang (2022) conclude that work-family initiatives have the potential to improve employee mental health, particularly among those who are experiencing high levels of work–family conflict. They argue that employers should consider implementing these initiatives as part of a broader strategy to promote employee well-being and reduce workplace stress. At present, few mixed methods studies have reported the experiences, stress overload, and influencing factors of nurse managers during the pandemic. Thus, it is unclear whether findings about front-line nurses’ experiences and adverse health reactions, such as psychological stress, extend to nurse managers. Therefore, it is necessary to explore the influencing factors of stress overload among nursing managers, which are different from those of front-line, using quantitative and qualitative research methods.

## Aims and hypotheses

This study aimed to develop a comprehensive understanding of stress load and its associated factors among nurse managers. The specific research questions were: (1) What is the level of stress load among nurse managers? (Quantitative); (2) What are the factors influencing stress load among nurse managers? (Quantitative); (3) What factors increase or reduce stress load among nurse managers? (Qualitative); and (4) To what extent do qualitative data on nurse managers’ perceived factors influencing stress load compensate for quantitative data about levels and factors affecting stress load? (Mixed method).

**Figure 1:**
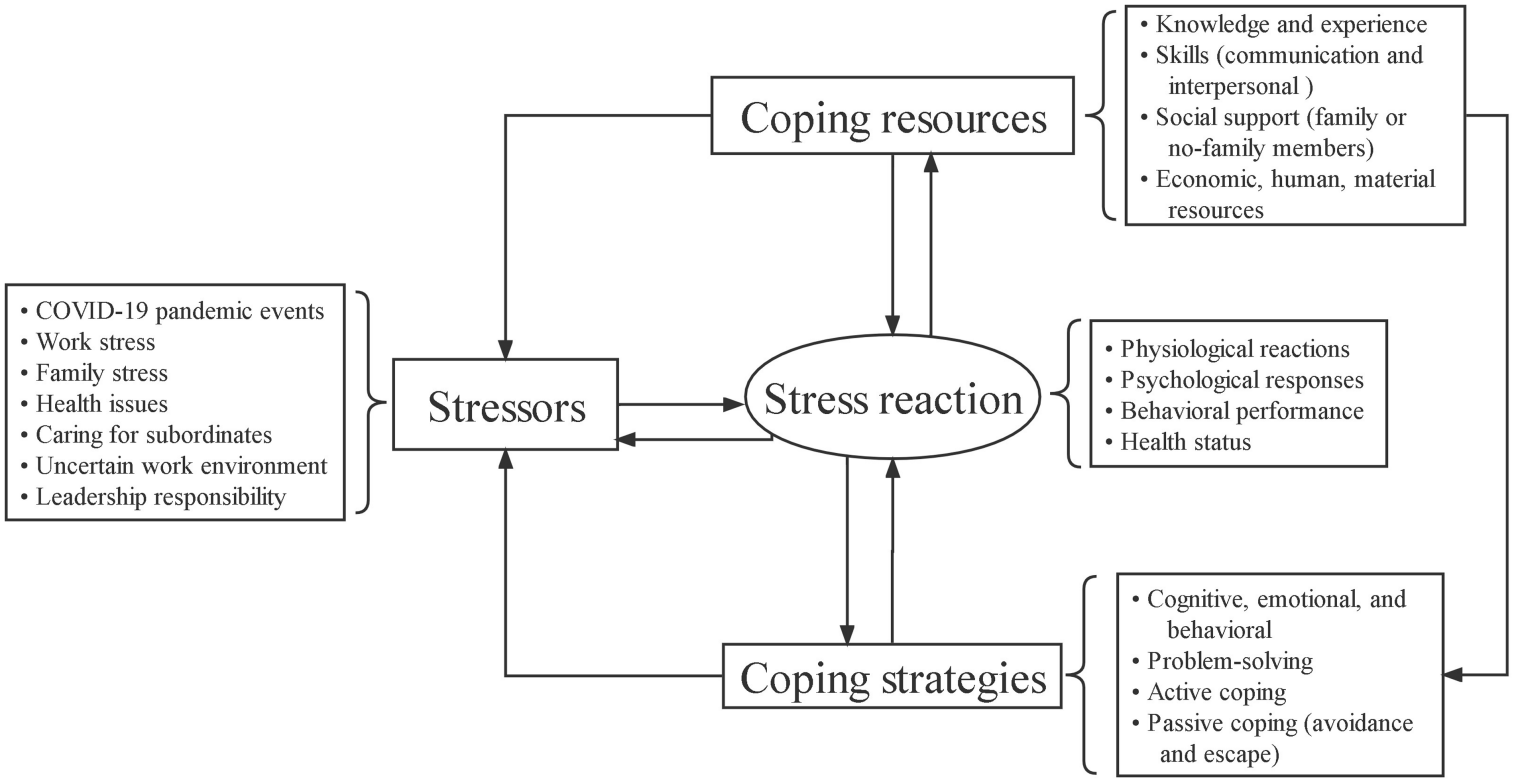
Theoretical model. Annotation: Stressors are comprised of both external and internal factors. Coping resources refer to various resources that individuals can utilize. Coping strategies refer to the ways and strategies that individuals use coping resources to deal with stress. Coping outcomes refer to an individual’s performance and consequences after coping with stress.

This study explores the relationships based on the framework of stress theory: (1) physical coping status, infectious disease work experience, staff knowledge about epidemic response, uncertain work environment, resource scarcity, and social support as potential predictors of stress overload for nurse managers (2); potential indicators of stress overload among nurse managers as determined through qualitative research; and (3) whether adequate support from families, employees, and superiors mediated the negative effects of high work demands on stress responses. The theoretical model is illustrated in Figure 1.

## Materials and methods

### Design

**Figure 2:**
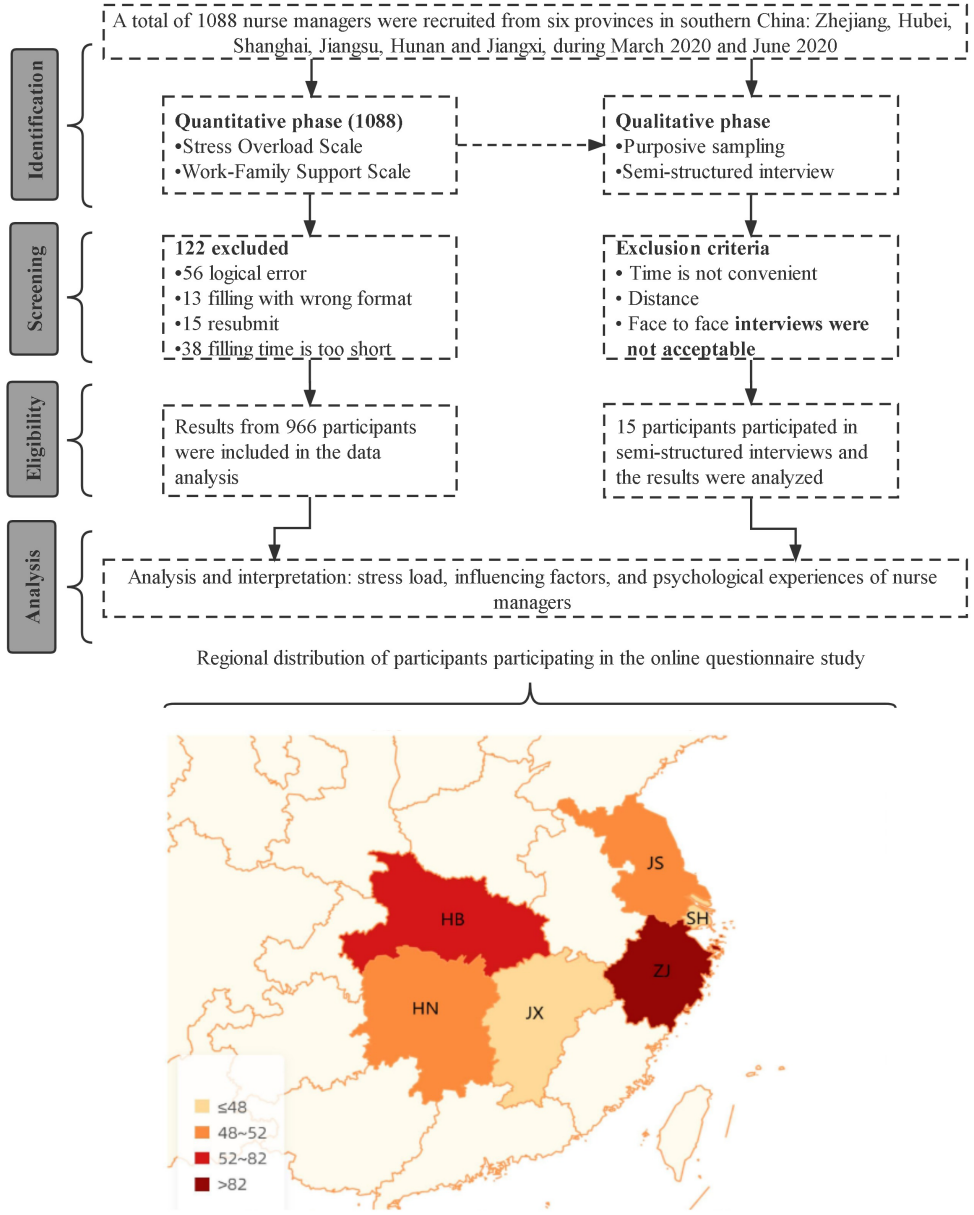
This study’s flowchart is as follows: Respondents from six provinces in southeast China were included, namely Zhejiang (ZJ), Hubei (HB), Shanghai (SH), Jiangsu (JS), Hunan (HN), and Jiangxi (JX). Most participants were from Zhejiang Province.

A sequential explanatory mixed methods design was adopted in this study. Quantitative data were collected and analyzed during the initial phase. This was followed by a second phase of qualitative data collection to further explain the findings obtained from the initial phase. The scale used in this study investigated factors associated with stress load among nurse managers and provided context and significance for the findings in light of the description of psychological burden that managers endured during the pandemic. This mixed methods design thus accounts for the strengths and weaknesses of the two approaches. This study followed the STROBE and COREQ checklists. A flowchart of participant recruitment is shown in Figure 2.

### Setting and sampling

Data for this study were collected from six provinces in southern China (Zhejiang, Hubei, Shanghai, Jiangsu, Hunan, and Jiangxi) during March 2020 and June 2020. Participants held managerial roles in regional hospitals, including head nurse of the ward, head nurse of the department, director of the nursing department, and associate dean of nurses.

The aim of this study was to investigate stress load among nurse managers during the pandemic. Preliminary survey results found that the incidence of moderate stress load was 57.31%. The necessary sample size was estimated to be 394 participants using PASS software, Version 15.0.5, based on a 95% confidence level, 5% tolerance error, 0.05 type I error, and two-sided intervals. Assuming 20% loss to follow-up, at least 493 participants were needed. As this study involved a cohort of 966participants, it exceeded the minimum sample size.

A non-random convenience sampling method was used to enroll participants. The purpose and methods of the study were explained to participants *via* an online network (WeChat or Ding Talk) or by email and their informed consent was obtained. The sample consisted of nurse managers who (a) had treated COVID-19 patients, (b) had participated in epidemic prevention and control work for at least 1 week, and (c) were willing to provide informed consent and voluntarily participate. Participants with obvious logical errors in their survey responses were excluded. After 15 interviewees were conducted qualitative interviews, the data reached saturation. The exclusion criteria are shown in Figure 2.

### Data collection

#### Quantitative phase

A personal information form was prepared by the researchers based on a literature review and group discussion (Mamais et al., 2022; Sansolo et al., 2022). The form contained 12 areas, including basic information such as hospital level, age, and history of training for public health emergencies.

#### Stress overload scale

Nurse managers’ stress overload was measured using the Stress Overload Scale (SOS), which was developed by Amirkhan (2012) and introduced, translated, and tested for reliability and validity by Su and Guo (2014). The scale includes 22 items in two dimensions: event load and individual vulnerability. Each item is rated on a 5-point Likert scale from 1 (never) to 5 (always). The overall score is based on the sum of all items and ranges from 22 to 110, with higher scores indicating more stress overload. The scale had excellent reliability and validity. The Cronbach’s alpha coefficient was 0.936, the content validity index (CVI) of the items was 0.86, and the CVI of each dimension was 0.80–0.86.

#### Work-family support scale (WFSS)

Work-family support was measured using the WFSS developed by Li and Zhao (2009). It comprises 30 items clustered into four factors: organizational support, leadership support, emotional support, and instrumental support. Items were scored on a 5-point Likert scale. Overall scores ranged from 30 to 150 points, with a higher score indicating higher support from work and family. The internal consistency coefficient of the questionnaire was 0.82 and the internal consistency coefficients of the four dimensions were 0.75, 0.78, 0.79, and 0.70, respectively. The correlation between the four factors was 0.37–0.56, while the correlation between the factors and total score was 0.57–0.85. The higher correlation between the factors and overall score indicated that the scale has excellent structural validity. In this study, Cronbach’s alpha coefficient of the WFSS was 0.944 and Cronbach’s alpha coefficient of each dimension was 0.902, 0.930, 0.879, and 0.840, respectively.

#### Qualitative phase

A phenomenological approach was used to conduct a qualitative study. Phenomenology provides the possibility to explore the change trajectory of nurse managers’ psychological stress (Udeagha et al., 2022; Odonkor and Yeboah, 2023). This approach helps ensure that respondents can effectively describe their experiences, thus providing deeper insight into their situation. Base on the reference “Qualitative Research Methods” and related qualitative studies, we designed the research flow chart Supplementary Figure 1.

#### Participant characteristics information of interviewees

General information about the interviewees was collected during the study. Basic characteristics, including age, gender, professional title, working years, etc., are provided in Supplementary Table 1.

#### Interview format

The interviews were completed by a researcher trained in qualitative research methods following a semi-structured interview format. The interview format was based on a literature research and group discussion. The interview format was tested in a pre-interview involving two nurse managers and then updated to address any shortcomings. The detailed interview questions are provided in Supplementary Table 2.

### Data collection process

#### Quantitative phase

WeChat was the main method used to contact responsible persons at each hospital on behalf of the nursing association to obtain their cooperation and briefly explain the survey. Professional network questionnaire software was used to generate electronic questionnaire links from the paper questionnaires. The purpose, significance, and related concepts of the questionnaire were explained using unified guiding language. The questionnaire took 10 min to complete. All questions were set as required items. To avoid repeated submission, the questionnaire could only be completed once by the same account, same device, and same IP address. All questionnaires were completed anonymously.

#### Qualitative phase

To ensure that the interviewees were representative, nurse managers in different roles were interviewed. Interviews were conducted until data saturation was reached (Manzo et al., 2022). To ensure privacy and confidentiality, interviews were conducted in specially arranged conference rooms. Interviews lasted on average 20 min. Before the interview, the purpose, significance, and process of the study were explained. The rights of the participants and principle of confidentiality were explained. Participants were clearly informed that the whole interview process would be recorded. Written informed consent was obtained from all participants. The researchers confirmed the commitment to confidentiality, no invasion of privacy, use of a number instead of the real name to ensure anonymity, and that the recordings would be destroyed after use. After each interview, the content was transcribed verbatim by two members of the research team Preliminary summary results (resulting thematic structure) were then returned to the participants in an electronic format for verification, confirming that their real experience was captured to ensure the accuracy of the findings.

#### Data analysis

Quantitative data were analyzed using SPSS 23.0 (IBM, United States) using descriptive statistics such as means and standard deviations (SD). Relationships between continuous variables were analyzed using Pearson correlation analysis. Multiple stepwise regression analysis was used to determine the relationship between the factors and outcome variable. Stepwise selection is an automatic variable entry procedure based on the statistical contribution and significance of factors in predicting the outcome variable. Using the total stress overload score as the dependent variable and the significant factors identified by the single factor analysis as the independent variables, multiple linear regression was then used to analyze the factors influencing stress load among nurse managers. For analysis of qualitative data, Colaizzi’s seven-step phenomenological analysis method was adopted (Tesfaw et al., 2022), as shown in Supplementary Table 3. After confirmation, the most representative connotation was summarized as the themes and several subthemes.

#### Validity, reliability, and rigor

The Lincoln and Guba (1985) standards were used to ensure methodological rigor and improve data reliability (Lincoln and Guba, 1985; Durante et al., 2022). For confirmability, to eliminate the subjective experience of the researchers, a literature review and pre-interview with two managers were performed before the study. During data collection, the interview content was recorded and written notes were taken. After each interview, the recording content and records were reexamined to verify their accuracy. To ensure credibility, researchers translated the recordings verbatim into transcripts within 24 h of the interviews. The final summary results were sent to all participants as an electronic document for confirmation and approval. Two independent researchers sorted, summarized, and coded the data and then provided the results to experienced researchers for confirmation. The final theme categorizations, citations, and justification were discussed until consensus was reached. Dependability was ensured through clear descriptions of the research background, sample data collection, and analysis methods, as guided by the Comprehensive Standards for Qualitative Research Reporting (COREQ). The COREQ Checklist appendix (Tong et al., 2007) is provided in Supplementary Table 4. The stress overload and work-family support questionnaires are both psychometrically validated scales with good internal consistency. A data integration strategy was used for mixed method analysis. Joint displays was used to visually present the data together to draw out new insights or inferences (Guetterman et al., 2015).

#### Ethical considerations

This study complied with the Declaration of Helsinki. The study protocol was approved by the Ethics Committee of Dongyang People’s Hospital. Informed consent was obtained from all participants before data collection. Participation in this study was entirely voluntary.

## Findings

### Quantitative findings

A total of 1,088 nurse managers were recruited for this study, of which 966 (88.79%) provided valid responses and were included in the analysis. As shown in Table 1, the proportion of secondary and tertiary hospitals was similar, accounting for 47.72% (461/966) and 52.28% (505/966) of hospitals, respectively. Most respondents (610, 63.15%) were aged 30–40 years old. Most had the professional title of nurse-in-charge (69.57%), while about 92% were nursing clinical directors and 5 of the nurse managers were deans in charge of nursing. Of all respondents, 89.65% were married with children, and about 14% were the only child of their families. The health status of nurse managers was mainly good, with only 0.83% reporting a poor health status. About 76% of nurse managers had participated in public health emergency training, and more than 90% had sufficient knowledge and experience to deal with infectious diseases. Most respondents acquired infectious disease prevention knowledge through their hospital or continuing education training. Only 14.08% (136/966) of nurse managers believed that hospitals were still not fully prepared to deal with epidemic prevention and control. Less than 30% of nurse managers had confidence in the protective ability of clinical front-line nurses.

**Table 1:** Demographic characteristics and univariate analysis results of participants (*n* = 966).

Variable	*n* (%)	SOS mean (SD)	t/F	Value of *p*
Hospital level			−0.234	0.815
Secondary	461 (47.72)	61.53 (16.41)		
Tertiary hospitals	505 (52.28)	61.77 (14.87)		
Age			4.428	0.012
30–40	610 (63.15)	60.60 (16.35)		
41–50	304 (31.47)	63.84 (14.32)		
50-	52 (5.38)	61.66 (15.62)		
Professional title			2.849	0.058
Nurse in charge	672 (69.57)	60.99 (15.90)		
Nurse deputy director	223 (23.08)	62.49 (14.45)		
Nurse director	71 (7.35)	65.28 (16.03)		
Clinical Position			2.271	0.104
Head nurse of ward	892 (92.34)	61.35 (15.75)		
Director/deputy director of nursing department	69 (7.14)	65.45 (13.78)		
Associate dean of nurse	5 (0.52)	64.00 (8.63)		
Marital and fertility status			0.267	0.790
Yes	866 (89.65)	61.60 (15.16)		
No	100 (10.35)	62.13 (19.17)		
Health status			24.888	<0.001
Worse	8 (0.83)	74.38 (17.39)		
General	183 (18.94)	68.28 (14.49)		
Good	775 (80.23)	59.96 (15.39)		
One-child Family			0.072	0.943
Yes	136 (14.08)	61.57 (16.50)		
No	830 (95.92)	61.67 (15.48)		
Participated in disaster and other public health emergency training			1.591	0.112
Yes	731 (75.67)	61.20 (15.21)		
No	235 (24.33)	63.06 (16.77)		
Whether there are relevant courses on infectious disease management and protection in clinical education			1.819	0.069
Yes	903 (93.48)	61.41 (15.61)		
No	63 (6.52)	65.11 (15.39)		
Participated in a hospital organization or continuing education program on the prevention of infectious diseases			3.224	0.003
Yes	933 (96.58)	61.43 (15.70)		
No	33 (3.42)	68.06 (11.44)		
Adequate knowledge and experience in the care of infectious diseases			3.310	0.001
Yes	899 (93.06)	61.20 (15.58)		
No	67 (6.94)	67.72 (14.89)		
Whether the hospital is fully prepared for the prevention and control of the sudden epidemic			5.582	<0.001
Unprepared	136 (14.08)	68.48 (14.88)		
Fully prepared	830 (85.92)	60.54 (15.46)		
As a manager, are you worried about the lack of knowledge of infectious disease protection of clinical nurses			−8.332	<0.001
Yes	688 (71.22)	64.23 (15.07)		
No	278 (28.78)	55.29 (15.12)		

SOS, stress overload scale score; SD, standard deviation.

The average stress overload score among all respondents was 61.66 (SD 15.62), and the score rate was 56.05%, indicating a moderate to high degree of stress. The average event load dimension score was 32.46 (SD 7.62). Of this items, the mean score for”feeling under too much stress and responsibility” was highest 3.76 (0.92), while the mean score for”feeling unmotivated to move forward” was the lowest 2.85 (0.92). The average dimension of individual vulnerability score was 29.20 (SD 9.29), of which the item “feeling things more than you can handle”had the highest score at 2.73 (0.85) and the item “I feel like my life was out of control”had the lowest score at 2.18 (0.99; Table 2). The work-family support score was 111.17 (SD 19.99) and the score rate was 74.11%, indicating that participants had a high level of support (Table 3).

**Table 2:** Stress overload among nurse managers.

Variable	Mean	SD	Score rate
Event load	32.46	7.62	64.92
Feeling under too much pressure and responsibility	3.47	0.98	
I feel that my heart was willing but not able to handle things	3.04	0.95	
Feeling nervous	3.11	0.95	
Feeling too busy to take a break	3.17	1.02	
Feeling tied down by responsibilities	3.41	1.06	
Feel that there was not enough time to deal with and finish something	3.31	0.97	
Feeling busy, rush about	3.43	0.98	
Feeling too much to do and not enough time	3.46	0.95	
Feeling that something was not going as expected	3.19	0.91	
I feel like I’m not motivated to do something	2.85	0.97	
Individual vulnerability	29.20	9.29	48.67
I feel like my life was out of control	2.18	0.99	
Feeling powerless	2.38	0.95	
I feel like everything is going wrong	2.39	0.88	
Feeling things go way beyond what you can handle	2.73	0.85	
Feeling that something was just too much to handle	2.55	0.84	
I feel like giving up when I encounter things	2.19	0.89	
I feel like there is so much going on, it was overwhelming	2.57	0.95	
I felt like something was really bad	2.22	0.90	
I felt like I was carrying a heavy burden	2.63	1.01	
I feel like I’m so unlucky.	2.31	0.95	
I feel like I had a lot on my mind	2.67	0.97	
I feel so stressed there was no escape	2.38	1.04	

Score rate (%) refers to the ratio of the mean value of a scale dimension to the total score of items in that dimension, expressed as a percentage.

**Table 3:** Below are the items with the highest and lowest scores in different dimensions of the family work support scale survey.

Variables	Mean	SD	Score rate
Organizational support	38.86	8.77	70.65
Organization always recognized for our job performance	3.86	0.89	
Units and organizations provided us with information about caring for the elderly and educating children	3.06	1.19	
Leadership support	32.85	7.19	73.00
When I have problems in my work, the leader will depend on the situation, not just criticize	3.83	0.86	
The leader understood me when family or personal issues interfere with my work	3.56	1.01	
Emotional support	23.67	4.29	78.90
My family always consoled me when I had problems at work	4.09	0.82	
My family often offered different opinions and opinions on my work problems	3.67	0.98	
Instrumental support	15.78	2.95	78.90
My family always do more housework when I am busy at work for a certain period of time	4.14	0.88	
My family was interested in what I was doing	3.69	0.95	

Score rate (%) refers to the ratio of the mean value of a scale dimension to the total score of items in that dimension, expressed as a percentage.

As shown in Table 4, work–family support was negatively related to stress overload among nurse managers (*r* = −0.551, *p* < 0.01). Furthermore, event load and individual vulnerability were negatively related to work-family support (*r* = −0.471, −0.539, respectively, all *p* < 0.01). Thus, stress load decreased as the degree of work-family support increased.

**Table 4:** Means, standardize deviations, and correlations.

Variables	Mean	SD	1	2	3
Event load	32.46	7.62			
Individual vulnerability	29.20	9.29	0.703^*^		
SOS	61.66	15.62	0.906^*^	0.938^*^	
FWSS	111.17	19.99	−0.471^*^	−0.539^*^	−0.551^*^

* *p* < 0.01 (two-tailed). FWSS, family work support scale; SOS, stress overload scale.

Event load and individual vulnerability are two dimensions of the stress overload scale.

Variables with statistically significant correlation in the univariate analysis were then used as independent variables, and stress load was used as the dependent variable, in the stepwise multiple linear regression analysis. As shown in Table 5, work-family support explained to 30.3% of variance, lack of knowledge of infectious disease to protect clinical nurses explained to 3.7% of variance, and health status contributed to 1.1% of the variance in stress load. Work-family support and health status were negatively associated with stress load (*β* = −0.551, −0.105, respectively, all *p* < 0.001). Lack of knowledge of infectious disease protection of clinical nurses was positively associated with stress load (*β* = 0.193, *p* < 0.001).

**Table 5:** Stepwise regression analysis of factors contributing to stress load among nurse managers.

Variables	*B*	S.E	Beta	*t*	Value of *p*	95% CI
Step 1
Family work support	−0.430	0.021	−0.551	−20.476	0.001	−0.471, −0.389
	*R*	*R^2^*	Adjusted *R^2^*	∆*R^2^*	∆*F*	*F*-value
	0.551	0.303	0.302	0.303	419.253	0.001
Step 2
Family work support	−0.411	0.021	−0.526	−19.941	0.001	−0.452, 0.371
Lack of knowledge of infectious disease protection of clinical nurses	6.657	0.910	0.193	7.316	0.001	4.871, 8.443
	*R*	*R^2^*	Adjusted *R^2^*	∆*R^2^*	∆*F*	*F*-value
	0.583	0.340	0.338	0.037	53.525	0.001
Step 3
Family work support	−0.395	0.021	−0.505	−18.884	0.001	−0.436, −0.354
Lack of knowledge of infectious disease protection of clinical nurses	6.529	0.904	0.189	7.225	0.001	4.755, 8.302
Health status	−3.877	0.977	−0.105	−3.967	0.001	−5.795, −1.959
	*R*	*R^2^*	Adjusted *R^2^*	∆*R^2^*	∆*F*	*F*-value
	0.592	0.350	0.348	0.011	15.736	0.001

S.E, standard error; FWSS, family work support scale; CI, confidence level.

*R*^2^, known as the coefficient of determination, that represents the proportion of the variance in the dependent variable that is explained by the independent variable(s) in a regression model.

Adjusted *R*^2^, adjusted coefficient of determination. The adjusted *R*^2^ provides a more accurate indication of the goodness of fit of the regression model, especially when the number of independent variables is large.

∆*R*^2^, known as the change in *R*^2^, that represents the increase in the coefficient of determination (*R*^2^) when an additional independent variable is added to a regression model. It measures the contribution of a specific independent variable to the explanation of the variance in the dependent variable after controlling for other independent variables in the model.

### Qualitative findings

#### Themes

**Figure 3:**
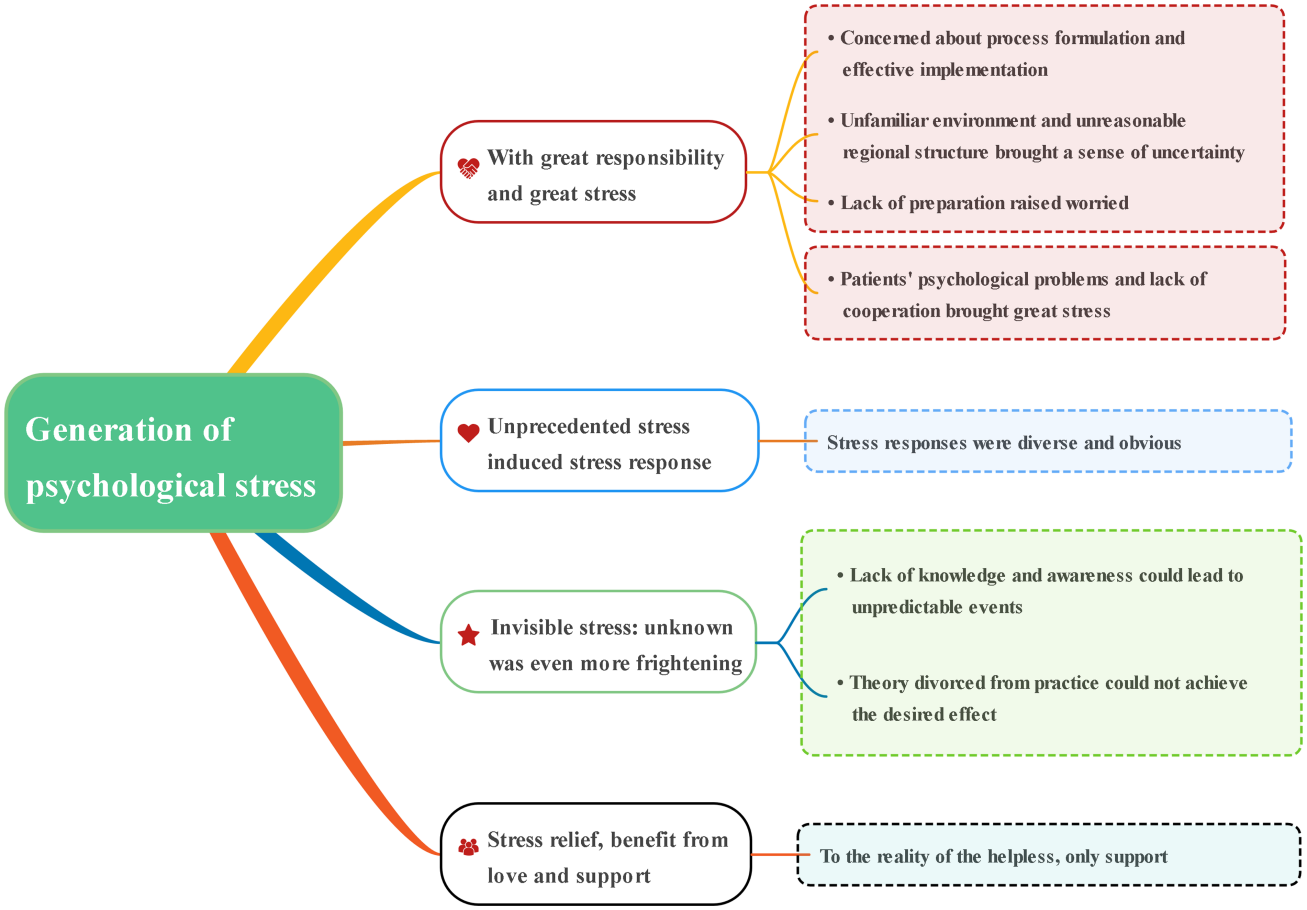
Theme and sub-theme.

Four main themes influencing stress load among nurse managers emerged from the interviews: great responsibility and great stress; unprecedented stress-induced stress response; invisible stress: the unknown was even more frightening; and stress relief from love and support. The sub-themes and quotes are presented in Figure 3 and Supplementary Table 5.

#### With great responsibility and great stress

Work stress is a harmful physical and emotional response caused by a mismatch between work overload and the abilities, resources, and needs of employees. High levels of stress can be attributed to over-commitment by nurse managers, leading them to take on excessive responsibilities and possibly increasing their work overload, subsequently leading to a high risk of vicarious trauma (D'Emeh et al., 2021). One nurse manager stated that:

“As head of the nursing department, I had been involved in decision-making throughout the pandemic. For me, planning and process decisions affect the overall outcome, and I felt a lot of responsibility and stress. Wearing and taking off protective clothing was a very important task: if it was not performed correctly, it could lead to cross-infection within the hospital. I was always worried about whether medical staff could seriously implement these new procedures. So I forced the entire staff to repeat these processes to make sure everything was right. I thought about these things every day, even in my dreams.”

Besides the development and effective implementation of processes during the pandemic, the work environment, regional structure, organizational readiness, staffing, and the internal fluctuations of patients in isolation were also sources of stress.

“A head nurse expressed that to prepare the transitional ward, we needed to start from scratch, and implement new systems and processes. I thought repeatedly about possible loopholes, whether prevention and control were reasonable, and the uncertainty brought by the unfamiliar environment. Some medical workers complained to me about the unreasonable division of the regional structure and the lack of isolation space. They were worried about cross-infection and infecting their family members.”

“At the beginning, we were not worried about protective equipment because we had enough supplies. However, as the epidemic spread and the number of patients increased, there was a serious shortage of supplies…, which caused great pressure. Patients also did not understand why they were isolated, …so their psychological problems become more serious and their emotions were high. They did not cooperate with isolation were prone to conflict, and showed weak awareness of protection and a lack of professional psychological comfort, from the head nurse of the fever unit.”

#### Unprecedented stress-induced stress response

Due to high levels of stress and anxiety, nurse managers are at risk of developing health-related problems and being unable to adapt. One nurse manager stated that:

“I thought I was a very strong person. H7N9 and SARS had never made me so anxious, but this epidemic brought unprecedented stress and I felt I could not resist it (laughter). Too busy, too tired, scratchy throat, dry cough, chest tightness, palpitations, and psychological stress induced the recurrence of the underlying disease. I needed to take metoprolo tablets to control palpitations. Constant vigilance led to weight loss, anxiety (hair loss), and insomnia.”

This unprecedented pandemic not only affected the health of front-line nurses, who were vulnerable to mental health problems such as anxiety, depression, emotional exhaustion, fear, and burnout, it also affected nurse managers. A head nurse told us:

“After I was quarantined my home was sealed, which brought great stress to my family. I felt deeply ashamed to know that (tears).

“The nursing sisters in our department were infected, which was my responsibility. I did not protect them. I was very sorry for them. When I thought of this, my heart was very painful. Because of the nursing workforce shortage, nurses could not stand it and complained why it was all the same people and not everyone else, this was moral kidnapping.”

#### Invisible stress: the unknown was even more frightening

The impact of care-related uncertainty during the pandemic was attributable to changing intervention protocols. If nurses lack up-to-date knowledge and information about this new disease, it could increase uncertainty and fear, resulting in strong negative emotions. One nurse managers shared that:

“Lack of protection knowledge among front-line nurses: although there was training, they could not understand its connotation, and the implementation effect was not good after training. There was a lack of practical experience: training was just like discussing stratagems on paper.”

“I was also concerned about the new fever transition unit, where the nature of patients' illnesses was complex (emergency, maternity, etc.) and patient severity did not match the capacity of nurses being deployed there. Poor risk identification of patients and inadequate measures by nurses may lead to adverse events.”

#### Stress relief and benefit from love and support

Excessive responsibilities at home and at work for nurse managers exacerbated their stress and burnout, leading to chronic health problems. However, support was identified an important strategy to relieve psychological stress. Support is positively correlated with self-efficacy and sleep quality, and high levels of support reduce feelings of burnout and fatigue (Xiao et al., 2020). One nurse manager stated that:

“Although my husband occasionally complained about my frequent overtime work and questioned my poor working ability, even if I came home late during the epidemic, he would not complain. He would be more supportive, such as obtaining braised supplements, to prevent reduced disease resistance, and send comfort food that nurses could share with each other to reduce stress.”

“My children and parents were also very worried (my son encouraged me to wear a mask to protect myself from infection through the video). My parents said that it was my career and they could do nothing about it-they could only silently support me and ease my worries.”

## Mixed methods analysis

The integration of qualitative and quantitative data provided greater insights into the factors related to stress overload among nurse managers. The quantitative results demonstrated that work-family support score, health status, and knowledge of infectious disease protection among clinical nurses were significantly associated with stress overload. Notably, age, continued education, protection experience, and hospital readiness were not associated with nurse manages’ stress overload. The qualitative themes confirmed the quantitative results and expanded upon them. The integration of data generated one confirmed finding and one expanded finding. The lack of protection for front-line nurses led to increased stress overload for nurse managers, which was mitigated by work-family concern, as supported by qualitative data (confirmed). Although quantitative data revealed the score among nurse managers with moderate stress overload, it could not identify the source of stress. However, qualitative analysis revealed several triggers that influenced stress load, namely, decision-making, unfamiliar environment, unreasonable regional structure, manpower and material shortage, and patients’ psychological states (expanded). Table 6 presents a joint display of factors affecting stress overload among nurse managers based on the integrated analysis.

**Table 6:** A joint display was created to summarize the factors affecting stress overload in nursing managers.

Quantitative	Qualitative	Mixed meta-inferences
Lack of knowledge of infectious disease protection of clinical nurses was positively associated with stress load (*β* = 0.193, *p* < 0.001) Work-family support and health status were negatively associated with stress load (*β* = −0.551, −0.105, respectively, all *p* < 0.001), with the model, the work-family support explained to 30.3% of the variance (*R*^2^ = 0.303, *p* < 0.001)	Unknown was even more frightening: Nurses lack up-to-date knowledge and information about this new disease, it could increase their uncertainty and fear, resulting in strong negative emotions. *“At the beginning, due to insufficient cognition of medical staff, patients were placed across each other, and inadequate protective measures resulted in cross infection.”* *“Front-line nurses received theoretical knowledge and operation training, but they could not flexibly apply the isolation principle, so they need to learn from practice (e.g., in order to prevent the tape of the goggles from being contaminated, they need to wear hats and prepare film gloves, and try to take every detail into consideration).”* Love and support: Support is an important strategy to relieve psychological stress. Low levels of support increased signs of compassion fatigue, burnout and moral exhaustion among front-line nurses. *“I could not go home to avoid risking infection to my family. Both children and parents were taken care of by my husband, so there was no family worry. Compared with the stress brought by the work, this was perhaps the most gratifying thing.”*	Confirmed. First-line primary nurses who had close contact with COVID-19 patients lack the knowledge and skills required for infectious disease protection, were unfamiliar with epidemic prevention procedures, and frequent changes in protection policies and guidelines had increased the management stress of nurse managers. On the contrary, the care and support from family and work could relieve the stress to some extent.
The source of the factors influencing the stress load had not been determined	Responsibility and stress coexist: High levels of stress could be attributed to over-commitment by nurse managers, leading them to take on excessive responsibilities. *“As head of the nursing department, I had been involved in decision-making throughout the pandemic. For me, planning, process decisions affect the overall outcome, feel a lot of responsibility and stress.”* Sense of uncertainty: Work environment, regional structure, organizational readiness and staffing, and the internal fluctuations of patients in isolation were also sources of stress. *“The area was not properly divided, medical staff cannot communicate effectively, there was not enough isolation space, and the fear of being infected leaded to cross infection between family members and medical staff.”* *“There was a serious shortage of supplies and nurses…, which brought us great stress. Patients with serious psychological problems, high mood, easy to friction.”*	Expanded. The source of stress was not known from quantitative data. However, the qualitative result showed that participation in decision-making, unfamiliar environment, unreasonable regional structure, manpower and material shortage, and patients’ psychological triggered stress load of nurse managers.

## Discussion

Few studies have examined the influence of COVID-19 on stress overload and mental health among nurse managers from the mixed perspectives of quantity and quality. In this paper, we aim to investigate the stressors and influencing factors of nursing managers during the COVID-19 pandemic, which is a complex and multidimensional issue. Quantitative and qualitative methods can provide different types of information and data. Quantitative methods can collect large amounts of data and help us identify general patterns and trends, for example, we can use scales to assess the stress levels of nursing managers. Although it can provide useful information, when it comes to issues involving personal experiences and feelings, quantitative methods are unable to deeply understand people’s experiences and perspectives, as well as the motivations and reasons behind them. Merely using quantitative methods may overlook many important details and information. However, qualitative methods can help us delve into and understand issues from the participants’ perspectives. For example, we can use in-depth interviews to understand the thoughts and actions of nursing managers when dealing with stress. This requires qualitative research to provide deeper understanding and insights to help us better identify the problems and challenges faced by nursing managers during the pandemic, as well as the specific actions they take when dealing with stress. We chose these two methods because they complement each other and can help us gain a deeper understanding of the specific circumstances faced by nursing managers. This study thus provides novel insights into these factors. Mixed analysis showed that nurse managers experienced a moderate to high level of stress overload. Although many factors contributed to their stress, the lack of self-protection knowledge among front-line nurses and negative support at work and from family were the main factors. The roles of these factors were supported by both qualitative and quantitative analysis, with qualitative results providing additional insights that cannot have been realized through quantitative analysis alone.

Stress had been identified as a precursor to various health challenges and is associated with changes in cognitive, behavioral, and emotional functioning that could limit decision-making (Dyess et al., 2018). Rapid mobilization of resources and decision-making require strong and innovative care managers, whose roles are highly complex and stressful in the context of a public health event (Labrague et al., 2018). A longitudinal survey of nursing leaders during the early days of the COVID-19 pandemic indicated that communicating and implementing changing policies were the most pressing challenges and caused nurse leaders to experience unhealthy emotion, burnout, and even consider leaving the nursing profession (Joslin and Joslin, 2020; Chipps et al., 2022). Dyess et al. (2018) found that nurse managers may become emotionally and cognitively exhausted when faced with such responsibilities; these seemingly endless responsibilities could lead to uncontrolled stress, fatigue, and possible burnout (Leiter and Maslach, 2009). The results of the present study showed that stress overload among nurse managers was mainly event load, such as “feeling under too much stress and responsibility; feel bound by various responsibilities,” reflecting the excessive external events, responsibilities, and stress they were subjected to during the early stages of the epidemic. These results are consistent with those of Middleton et al. (2021), who found that managing critical situations and responding to rapidly changing guidelines was a challenge for mid-level nurse managers. This effect had myriad causes and stressors, including unpredictable staffing and protective equipment, complexity of roles, high workload, time pressure, moral dilemmas, uncertainty about job demands, concern for family members, and exposure to infection, as well as various other factors like increased anxiety, burnout, and self-fear. This might be related to the fact that most clinical nurses had not participated in systematic disaster emergency training, nor had they participated in public health emergencies, and thus lacked experience dealing with sudden infectious diseases. Although the hospital provided relevant knowledge and protection training, besides the nurses in the infection department, most clinical nurses had insufficient knowledge and protection measures for infectious diseases. The results of qualitative interviews in this study revealed that nurse managers with higher levels of anxiety tended to use maladaptive coping strategies, such as denial, self-blame, or behavioral disengagement. This finding is consistent with a study by Middleton et al. (2021). Maladaptive coping strategies are problematic, have a negative impact on mental health, and may lead to social isolation (Thompson et al., 2010). These effects may be related to nurses who move into middle management roles but are ill-prepared to cope with the demands of that role, as well as the added stress of the pandemic, ultimately leading to maladaptive coping strategies (Middleton et al., 2021). These findings are consistent with the literature on nursing managers responses to SARS, which points to an imbalance between crisis and response strategies (Lau and Chan, 2005).

The overall roles of nurses in disaster preparedness and responses have been recognized worldwide. Nevertheless, nurses often feel ill-equipped to respond to disasters (Veenema et al., 2016; Labrague et al., 2018). Middleton et al. (2021) suggested that high anxiety among nurse managers may be due to the intensity and lack of predictability of COVID-19. Even senior nurse leaders had never experienced such a crisis like this new and emerging threat, and the lack of knowledge about it led to a heavy workload. Unfortunately, nurse managers might not be trained or prepared to take on this additional burden (Dimino et al., 2021). A lack of exposure to infectious diseases during epidemics and pandemics, while ensuring that staff are well educated and supported, will only increase anxiety and stress for nurse managers. Although nurses completed training before entering the isolation ward, the breadth and depth of the training content were insufficient. Such insufficient knowledge among nurses would reduce self-efficacy, increase stress, and directly affect patients’ medical experience. This study showed that nurse managers’ concerns about protecting of clinical nurses were the main predictor of stress overload. Research has shown that clinical nurses often adopt negative attitudes and behaviors to deal with emergencies arising due to a lack of experience or their own ability to manage, resulting in reduced confidence and increased panic (Sun et al., 2021; Huff et al., 2023). Front-line nurses’ ability to cope with stress was positively correlated with professional attitudes, fear of contagion, emergency preparedness, and confidence responding to COVID-19 (Cui et al., 2021). Mehri et al. (2022) found that among nurses, especially those in the ICU, increased workload is an important cause of stress and also increases cognitive failure and decreases the quality of care and patient safety. The qualitative interview results of the present study indicated that “the newly established fever transition ward had a wide range of patients and complex disease nature (emergency rescue, puerpera, etc.). However, the severity of the ward was not in line with the ability to allocate qualified nurses. Risk identification of patients was poor and measures were not in place, potentially leading to adverse events.” This description is consistent with research by Wolf et al. (2017) and Rostami et al. (2021) who found that if nurses’ abilities do not match their working conditions, this can cause work stress and increase cognitive failure. Nurses in poor health will not be able to provide better physical and mental care to patients, ultimately increasing errors and occupational accidents. Callus et al. (2020) showed that by fully enacting and strengthening self-care interventions for front-line nurses, the related stress can be reduced.

Social support is a means of reducing stress and improving health and well-being and a popular concept in psychological research (French et al., 2018). Work-family specific support plays a central role in an individual’s experience of work–family conflict. A lack of social support is most likely to influence work–family conflict, causing work roles to interfere with family roles. Kossek et al. (2011) showed that a supervisor’s work-family support contributes to employees’ ability to simultaneously manage work and family relationships. Basis on a previous study about SARS, supports from supervisors and colleagues was the main predictor of reduced psychological impact (Chan and Huak, 2004). This study showed that work-family support as a major predictor was negatively correlated with stress overload. The results thus show that a high work–family support score indicates a high degree of support. This was related to the fact that, since the outbreak of the epidemic, the media positively promoted relevant knowledge and the public highly praised front-line medical workers, which indirectly increased support for work and family. As shown by French et al. (2018), social support may have a direct relief effect on stressors, or social support may directly relieve stress. The results of this study found that, compared to general support from the organization and leadership dimensions, emotional support and instrumental support from family members had a higher scored, indicating that family support was a strong motivation for nurse managers to work. Emotional support refers to resources directed towards the receiver and self-evaluation of support, such as love, care, and trust. Instrumental support provides tangible resources, such as time or money, that can be used to directly manage stress (French et al., 2018). with family affairs and indirectly reduced stress load. Li et al. (2021) show that while family members provided the most support across all domains, non-family members also provided significant support, especially in the areas of instrumental support and appraisal support. However, the authors note that non-family members tended to provide less emotional and informational support compared to family members. Lapierre and Allen (2006) predicted that emotional and instrumental support would reduce stress; however, each type of support provides unique resources. Emotional support provides resources to relieve stress caused by psychological factors, while instrumental support provides tangible resources and help that directly alleviates stress (French et al., 2018). One study found that instrumental support, which provided tangible resources to reduce conflict, may be the most effective way to reduce time-based conflict and was more predictive of work–family conflict decisions than emotional support (Shockley and Allen, 2013). According to conservation theory, stress occurs when resources are lost or threatened or when expected resources do not materialize, which in turn is associated with negative psychological outcomes, such as depression (O'Brien et al., 2014). Spousal support was a predictor of depression and moderated various relationships from work–family conflict to depression. Emotional and instrumental support from spouses was positively associated with the mental health of working mothers. Cultural values and norms undoubtedly influence the extent to which spouses share responsibilities related to family (O'Brien et al., 2014). Our qualitative results support this perspective: family members were accustomed to the job characteristics of nurse managers, given their job responsibilities and requirements. Overall, from the point of view of time and energy, family support alleviated nurse managers’ worries about dealing.

## Limitations

Although this study has provided some novel perspectives on stress overload and psychological experiences among nursing managers, there are some limitations. First, this study provided a descriptive and cross-sectional view of factors affecting nurse managers, however, the data source was related to only one point in time, and the complexity and evolution of these factors may not have been captured, leading to uncertainty about the causal relationships between variables. Second, recall bias may have influenced the results. Since sample selection in convenience sampling is based on convenience, the sample may not represent the entire population, which can result in selection bias that can affect the accuracy of the results. Moreover, the sample selected by convenience sampling is typically non-random, making it difficult to generalize the results to the entire population.

There are differences in the distribution of medical resources, human resources, and educational resources in different provinces and regions of mainland China. This study investigated the stress burden and influencing factors faced by nursing managers in six southern provinces of China (Zhejiang, Hubei, Shanghai, Jiangsu, Hunan, Jiangxi) during the COVID-19 pandemic, mainly based on convenience or availability of the sample. When selecting the study area, we mainly considered that the southern region had a more severe epidemic situation, and nursing managers in the southern region may have special situations and stress in some aspects. Although this study cannot represent nursing managers in all of China, the results obtained are based on the situation of nursing managers in the study areas, and these nursing managers may face different problems and stress from those in other areas. This means that the findings of this study are based on special circumstances and regions and cannot be generalized to the entire population, but the findings can provide reference for other regions.

In future studies, more rigorous sampling methods such as random or systematic sampling could be used to obtain more representative samples, reducing the potential for bias and improving the generalizability of the findings. Additionally, longitudinal study designs could be employed to investigate the changes in stress and influencing factors over time, providing more valuable information about the fluctuations of these factors over time.

## Conclusion

Meeting staff protection needs and achieving organizational goals is a matter of priorities, emotions, and reprioritization. Trying to resolve associated discordant processes and maintain a “balance” between them can expose nurse managers to significant hidden stress and psychological challenges, which may affect their ability to cope and commit to the organization. Therefore, during disaster and emergency situations, senior hospital leaders should plan and provide relevant training for nurse managers and develop employee family benefits that aim to reduce anxiety levels and address maladaptive coping strategies. Regarding how to relieve the stress load of nurse managers, future research should establish multi-dimensional intervention measures and plans and identify personalized solutions based on the factors identified in this study.

## Data availability statement

The original contributions presented in the study are included in the article/Supplementary material, further inquiries can be directed to the corresponding author.

## Ethics statement

This study involving human participants were reviewed and approved by the Clinical Research Ethics Committee of the Affiliated Dongyang Hospital, Wenzhou Medical University. Written informed consent was obtained the individual(s) for the publication of any potentially identifiable images or data included in this article.

## Author contributions

HW, FC, and YJ made substantial contributions to conception and design. RW and SC collected and collated the data. FC and LH were responsible for analyzing and interpreting the data. FC and MY were involved in drafting the manuscript or revising it critically for important intellectual content. All authors contributed to the article and approved the submitted version.

## Conflict of interest

The authors declare that the research was conducted in the absence of any commercial or financial relationships that could be construed as a potential conflict of interest.

## Publisher’s note

All claims expressed in this article are solely those of the authors and do not necessarily represent those of their affiliated organizations, or those of the publisher, the editors and the reviewers. Any product that may be evaluated in this article, or claim that may be made by its manufacturer, is not guaranteed or endorsed by the publisher.
