# Supplementary material for: Stress overload, influencing factors, and psychological experiences of nurse managers during early stages of the COVID-19 pandemic: a sequential explanatory mixed method study
Source: Front Psychol. 2023 Jun 30;14:1187433. doi: 10.3389/fpsyg.2023.1187433 (PMC10348901; doi:10.3389/fpsyg.2023.1187433)
Supplement: Supplementary file 1 [file Data_Sheet_1.docx]

Supplementary Material

## Supplementary Figures and Tables


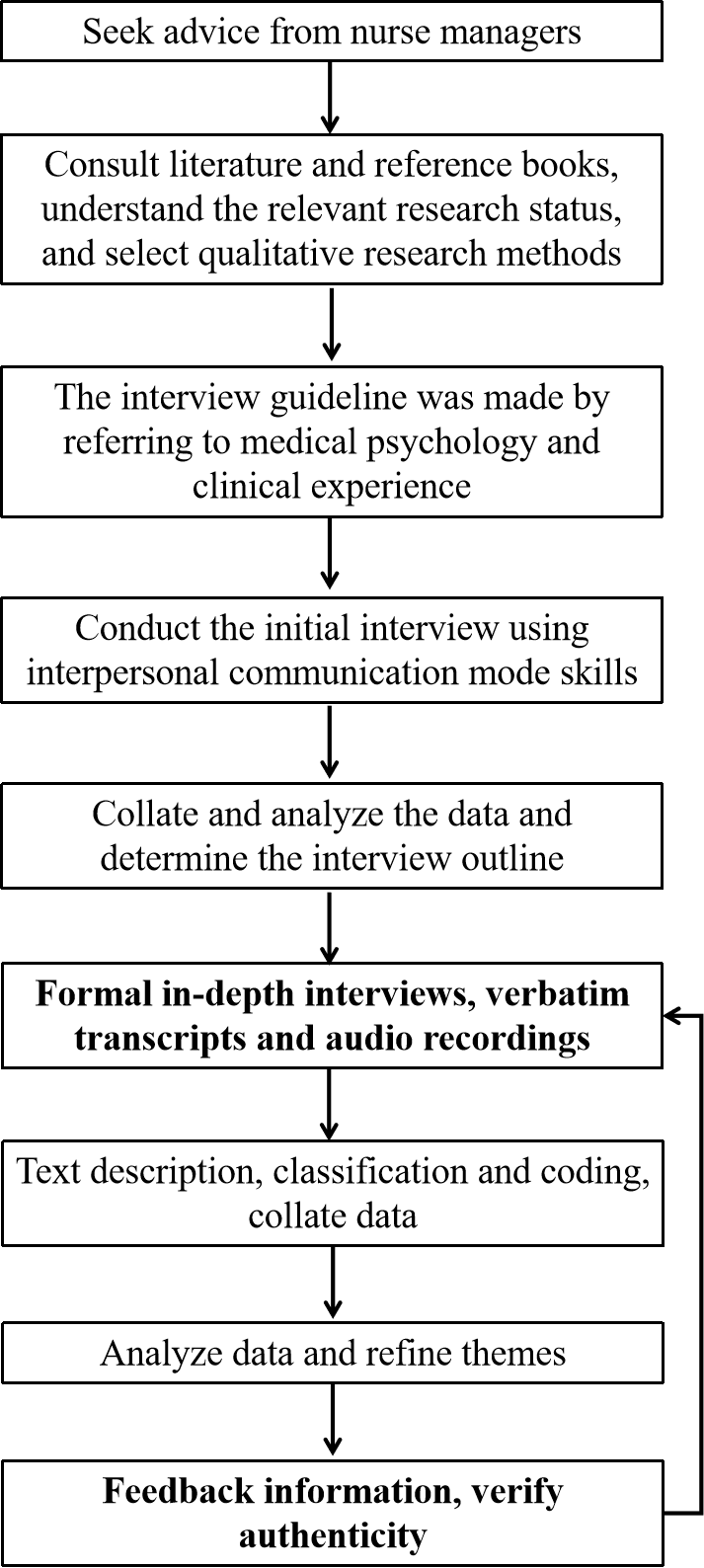


**Supplementary Figure 1.** Flow chart of qualitative research.

**Supplementary Table 1.** Interviewees’ characteristics

| **No** | **ID** | **Gender** | **Age** | **Professional title** | **Clinical Position** | **Working years** |
| --- | --- | --- | --- | --- | --- | --- |
| 1 | HM1 | Female | 49 | Nurse director | Associate dean of nurse | 30 |
| 2 | HM2 | Female | 44 | Nurse deputy director | Director of nursing department | 27 |
| 3 | HM3 | Female | 56 | Nurse director | Head nurse of ward | 38 |
| 4 | HM4 | Female | 44 | Nurse deputy director | Head nurse of ward | 27 |
| 5 | HM5 | Female | 47 | Nurse deputy director | Head nurse of ward | 30 |
| 6 | HM6 | Female | 49 | Nurse director | Head nurse of ward | 32 |
| 7 | HM7 | Female | 48 | Nurse deputy director | Head nurse of ward | 32 |
| 8 | HM8 | Female | 38 | Nurse in charge | Head nurse of ward | 16 |
| 9 | HM9 | Female | 50 | Nurse deputy director | Head nurse of ward | 33 |
| 10 | HM10 | Female | 48 | Nurse deputy director | Head nurse of ward | 30 |
| 11 | HM11 | Female | 50 | Nurse director | Deputy director of nursing department | 33 |
| 12 | HM12 | Female | 43 | Nurse deputy director | Head nurse of ward | 26 |
| 13 | HM13 | Female | 42 | Nurse deputy director | Head nurse of ward | 26 |
| 14 | HM14 | Female | 38 | Nurse in charge | Head nurse of ward | 18 |
| 15 | HM15 | Female | 36 | Nurse in charge | Head nurse of ward | 15 |

**Supplementary Table 2.**  Interview guide.

| **Number** | **Questions** |
| --- | --- |
| 1 | Please described in detail what makes you feel stressed? |
| 2 | What were the symptoms or reactions of the front-line nurses that make you feel unusually anxious? |
| 3 | What do you find most gratifying about the support from your family? |
| 4 | Do you have anything else to add or share? |

**Supplementary Table 3.** Phenomenological analysis of seven-steps procedure and description

| **Procedure** | **Description** |
| --- | --- |
| **Step 1**  Familiarization | The researchers were fully familiar with and understood all the information provided by the study participants through repeated and careful reading of the collected data |
| **Step 2**  Identifying significant statements | Analyze the data word by word to identify and extract important and meaningful statements related to the research question |
| **Step 3**  Formulating meanings | Researchers construct/encode for recurring ideas. But try to "bracket" your existing presuppositions related to the phenomenon. |
| **Step 4**  Clustering themes | The coded ideas are pooled to find meaningful common concepts. Form the theme rudiment; At this time, you still need to "bracket" yourself existing ideas or experiences, especially theoretical knowledge from the literature. |
| **Step 5**  Developing an exhaustive description | Researcher need to provide a detailed description of each themes generated in Step 4, and to select and quote the original statements from the participants. |
| **Step 6**  Producing the fundamental structure | Compare similar themes and their descriptions together repeatedly to identify and extract similar ideas; then construct a short phrase that condenses the meaning of the themes. |
| **Step 7**  Seeking verification of the fundamental  structure | The resulting themes structure were returned to the participants for verification, asking if it captures their true experience, and ensuring the accuracy of the results. If there were a bias, the researcher must start from the first step and re-analyze the analysis step by step. |

**Supplementary Table 4.** Consolidated criteria for reporting qualitative studies (COREQ): 32-item checklist

| **No** | **Item** | **Guide questions/description** |  |
| --- | --- | --- | --- |
| **Domain 1**  **Research team**  **and reflexivity** |  |  |  |
| Personal Characteristics |  |  |  |
| 1 | Interviewer/facilitator | Which author/s conducted the interview or focus group? | Fei fei Cui |
| 2 | Credentials | What were the researcher's credentials? *E.g. PhD, MD* | Master of Nursing |
| 3 | Occupation | What was their occupation at the time of the study? | Nurse clinician |
| 4 | Gender | Was the researcher male or female? | Male |
| 5 | Experience and training | What experience or training did the researcher have? | He has been engaged in a variety of health-related research projects, nurses' behavioral intentions, self-efficacy management, pressure ulcers, diabetes, venous ulcers and other chronic wound management, and other clinical teaching for the past 5 years. He is also EWMA wound therapist and core member of the hospital's evidence-based care team. Participated in many online or offline continuing education training courses on qualitative and quantitative research. |
| Relationship with participants |  |  |  |
| 6 | Relationship established | Was a relationship established prior to study commencement? | Yes. A relationship was established in the first phase of the study. |
| 7 | Participant knowledge of the interviewer | What did the participants know about the researcher? e.g. personal goals, reasons for doing the research | After introducing the purpose of the study, participants knew that their participation was to help explore the psychological stress and inner feelings of nurse managers during the outbreak of the epidemic, so as to provide reference for the subsequent development of corresponding countermeasures |
| 8 | Interviewer characteristics | What characteristics were reported about the interviewer /facilitator? e.g. Bias, assumptions, reasons and interests in the research topic | The interviewer, based on previously  established relationship with participants, provided a conducive and trusting environment for participants to share their experiences. |
| **Domain 2**  **Study design** |  |  |  |
| Theoretical  framework |  |  |  |
| 9 | Methodological orientation and Theory | What methodological orientation was stated to underpin the study? *e.g. grounded theory, discourse analysis, ethnography, phenomenology, content analysis* | Phenomenology  Supplementary Table 1 |
| Participant  selection |  |  |  |
| 10 | Sampling | How were participants selected? e.g. purposive, convenience, consecutive,snowball | Purposive sampling based on  participants’ availability and willingness to  participate in the second phase of the study  Page 4, Line 145-152 |
| 11 | Method of approach | How were participants approached? e.g. face-to-face, telephone, mail, email | Participants were interviewed via face to face  after they had completed the quantitative phase of the study  Page 6, Line 202-213 |
| 12 | Sample size | How many participants were in the study? | 15 Participants  Page 4, Line 150-151 |
| 13 | Non-participation | How many people refused to participate or dropped out? Reasons? | During the pandemic, 30 participants were unable to participate in interviews due to inconvenient time and distance |
| Setting |  |  |  |
| 14 | Setting of data  collection | Where was the data collected? e.g. home, clinic, workplace | Workplace  Page 6, Line 202-213 |
| 15 | Presence of non- participants | Was anyone else present besides the participants and researchers? | Yes, research assistants |
| 16 | Description of sample | What are the important characteristics of the sample? e.g. demographic data, date | The sample consisted of nurse managers in various roles who had consented and participated in the first phase of the study  Page 6, Line 202-203 |
| Data collection |  |  |  |
| 17 | Interview guide | Were questions, prompts, guides provided by the authors? Was it pilot tested? | Interview guides consisted of open ended questions with prompts and follow up questions. The guide was pretested and modified before use  Page 5, Line 187-191；Page 6, Line 202-213 |
| 18 | Repeat interviews | Were repeat interviews carried out? If yes, how many? | No |
| 19 | Audio/visual recording | Did the research use audio or visual recording to collect the data? | The data was audio recorded with the consent of participants.  Page 6, Line 206-207 |
| 20 | Field notes | Were field notes made during and/or after the interview or focus group? | Field notes were made during the interview, after the interview, the records were sorted out and checked |
| 21 | Duration | What was the duration of the interviews or focus group? | The interviews were mostly between 20- 30 minutes |
| 22 | Data saturation | Was data saturation discussed? | Yes |
| 23 | Transcripts returned | Were transcripts returned to participants for comment and /or correction? | Yes |
| **Domain 3**  **Analysis and findings** |  |  |  |
| Data analysis |  |  |  |
| 24 | Number of data coders | How many data coders coded the data? | Two |
| 25 | Description of the coding tree | Did authors provide a description of the coding tree? | No |
| 26 | Derivation of themes | Were themes identified in advance or derived from the data? | Themes were derived from the data |
| 27 | Software | What software, if applicable, was used to manage the data? | Not applicable |
| 28 | Participant checking | Did participants provide feedback on the findings? | Yes |
| Reporting |  |  |  |
| 29 | Quotations presented | Were participant quotations presented to illustrate the themes / findings? Was each quotation identified? e.g. participant number | Participants’ quotations were presented  to illustrate the themes and findings.  Each quote was identified by the  participant number |
| 30 | Data and findings  consistent | Was there consistency between the data presented and the findings? | There was consistency between the data  presented and the findings. |
| 31 | Clarity of major themes | Were major themes clearly presented in the findings? | Major themes were clearly presented in  the findings Page 8-9, Supplementary Table 5 |
| 32 | Clarity of minor themes | Is there a description of diverse cases or discussion of minor themes? | Minor themes were adequately discussed  Supplementary Table 5；Table 6 |

**Supplementary Table 5.** Themes and examples of quotes from the thematic analysis

| **Main themes** | **Sub-themes** | **Quotations** |
| --- | --- | --- |
| With great responsibility and great stress | Concerned about process formulation and effective implementation | •“Planning, process decisions affect the overall outcome, feel a lot of responsibility and stress”.  •“Worried that the new process could not be implemented seriously, all the staff repeatedly simulated the process, such as putting on and taking off protective clothing”  •“If the protection process was not implemented in place, it could lead to nosocomial cross infection.”  •“The process of removing the mask for glasses wearers was not properly performed, and the movement of pushing glasses was easy to cross infection”  •“The hospital infection department conducted standard protection knowledge training, and the inspection team checked whether the protection process was reasonable.” |
|  | Unfamiliar environment and unreasonable regional structure brought a sense of uncertainty | •“To prepare the transitional ward, we need to start from scratch, implement new systems and processes, and think repeatedly about the possible loopholes, whether the prevention and control was reasonable, the uncertainty brought by the unfamiliar environment.”  •“The area was not properly divided, medical staff cannot communicate effectively, there was not enough isolation space, and the fear of being infected leaded to cross infection between family members and medical staff.”  •“After regional division, supervision is not in place (the purpose of installing monitoring is to urge good protection, protect themselves, their families and patients), and how to protect places where monitoring cannot reach is unknown.” |
|  | Lack of preparation raised worried | •“The protective materials were not worried at first, but with the spread of the epidemic and the increase of patients, the whole country became worried after the shortage of materials.”  •“N95, protective clothing materials were insufficient, industrial protective clothing could not be used to seal the face, could only be used with tape, easy to pollution when taking off.”  •“There was no visual system in the negative pressure area, so we can only communicate through wechat and phone.” |
|  | Patients' psychological problems and lack of cooperation brought great stress | •“Patients did not understand and cooperate with isolation, and their awareness of protection was weak.”  •“Patients with serious psychological problems, high mood, easy to friction, lack of professional psychological comfort.” |
| Unprecedented stress-induced stress response | Psychological stress responses were diverse and obvious | •“During the epidemic period, too busy, too tired, scratchy throat, dry cough, chest tightness, palpidation, psychological stress induced the recurrence of the underlying disease, need to take metoprolo tablets to control palpidation.”  •“I think I am a very strong person. H7N9 and SARS had never been so anxious, but this epidemic had brought unprecedented stress and I felt that I cannot resist it.” ***(Laughter)***  •“After I was quarantined, my home was sealed, which brought great stress to my family. I feel deeply ashamed to know that. Constant vigilance leads to weight loss, anxiety (hair loss), insomnia.”  •“Nursing staff could not hold up for a long time (moral kidnapping) leading to complaints (why were all the same people, not others resting).”  •“Anxiety and physical discomfort caused by fear of infection of nursing staff: fear of infection of medical staff will lead to the spread of the epidemic. If nursing staff were infected, they may not be able to get through the whole life, resulting in anxiety and insomnia.” |
| Invisible stress: unknown was even more frightening | Lack of knowledge and awareness could lead to unpredictable events | •“The newly established fever transition ward in the hospital had a wide range of patients and complex disease nature (emergency rescue, puerpera, etc.). The severity of the ward was not in line with the ability of allocating nurses. The nurses' protection was qualified, but the risk identification of patients was poor and the measures were not in place, which may lead to the occurrence of adverse events.”  •“At the beginning, due to insufficient cognition of medical staff, patients were placed across each other, and inadequate protective measures resulted in cross infection.” |
|  | Theory divorced from practice could not achieve the desired effect | •“Before entering the isolation ward, front-line nurses received theoretical knowledge and operation training, but they could not flexibly apply the isolation principle, so they need to learn from practice (e.g., in order to prevent the tape of the goggles from being contaminated, they need to wear hats and prepare film gloves, and try to take every detail into consideration).”  •“Lack of protection knowledge among front-line nurses: although there was training, they could not understand its connotation, and the implementation effect was not good after training. Lack of practical experience: training is just like discussing stratagems on paper.” |
| Stress relief, benefit from love and support | To the reality of the helpless, only support | •“Being able to work in the nursing management position was closely related to the support of family members. Although they often work overtime at ordinary times, they may occasionally complain about their poor working ability, but during the epidemic, they will not complain even if they return home late, but they will be more supportive, such as braising supplements to prevent the decline of resistance.”  •“Although my husband is worried, he is not willing to apply pressure and only emphasizes protection. The children were also worried and did not tell their parents (the parents were unable to call and were very worried about it, but there was nothing they could do about it. This is your profession, so you should take good protection). Husband delivers love meal, nursing staff drink together, reduce stress through food.”  •“My husband thought that this is the duty of nurses, considering the risk of cross-infection, so it was not convenient to go home. The child was taken care of by his husband and parents, and the child was worried by video chatting with him at night. (Comfort himself to be careful, wear a mask, protect himself, and avoid infection）.” |
